# A novel tool for predicting the risk of cancer-specific early death in older patients with primary malignant melanoma of skin: a population-based analysis

**DOI:** 10.3389/fonc.2024.1387014

**Published:** 2024-09-06

**Authors:** Yan Lei, Shucui Wang, Jun Chen, Lanjun Liu, Linting Huang, Xiujuan Wu, Hui Xu, Yali Yang

**Affiliations:** ^1^ Department of Dermatology, Ninth People’s Hospital Affiliated to Shanghai Jiao Tong University School of Medicine, Shanghai, China; ^2^ Department of Dermatology, Children’s Hospital of Fudan University, National Children’s Medical Center, Shanghai, China; ^3^ Department of Laser Cosmetology, the Ninth People's Hospital Affiliated to Shanghai Jiao Tong University Medical College, Shanghai, China; ^4^ Department of Dermatology, Shanghai Xuhui Central Hospital, ZhongShan-Xuhui Hospital, Fudan University, Shanghai, China

**Keywords:** cancer-specific early death, nomogram, older, melanoma, skin, SEER

## Abstract

**Background:**

Primary malignant melanoma (MM) of skin threatens health, especially in the older population, causing a significant risk of early death. The purpose of this study was to establish a diagnostic nomogram to predict the early mortality risk in older patients with primary skin MM and to determine the independent risk factors of cancer-specific early death in such patients.

**Methods:**

The Surveillance, Epidemiology and End Results (SEER) database provided the clinical and pathological characteristics of older patients with primary skin MM from 2000 to 2019. Initially, a 7:3 random assignment was used to place the recruited patients into training and validation cohorts. Then, the independent risk variables of cancer-specific early death in those individuals were determined using univariate and multivariate logistic regression analysis. Those patients’ diagnostic nomograms were constructed using the acquired independent risk variables. Ultimately, the performance of the newly created diagnostic nomogram was verified using calibration curves, receiver operating characteristic (ROC), and decision curve analysis (DCA) curves.

**Results:**

In this study, 2,615 patients in total were included. Age, histology, liver metastasis, tumor stage, surgery, therapy, and radiation were found to be independent risk factors following statistical analysis, with a special emphasis on early death in older patients with primary skin MM. A diagnostic nomogram for the cancer-specific early death risk was created and validated based on these variables. High agreement was reported between the expected and actual probabilities in the calibration curves. Area under the curves (AUC) of the novel created diagnostic nomogram was greater than that of each independent risk factor, with AUCs for the training and validation cohorts being 0.966 and 0.971, respectively. The nomogram had a high value for its applicability in clinical settings, according to DCA.

**Conclusion:**

In older patients with primary skin MM, the current study created a diagnostic nomogram to predict the probability of cancer-specific early death. Because of the nomograms’ good performance, physicians will be better able to identify older patients who are at a high risk of early death and treat them individually to increase their survival benefit.

## 1 Introduction

Malignant melanoma (MM) is one of the most dangerous malignant neoplasms origins from epidermal melanocytes and is associated with a high risk of death (1). Based on the most recent cancer data released by the American Cancer Society on January 17, 2024, melanoma of the skin is the fifth most prevalent malignant tumor globally. In 2024, it is estimated that there will be 97,160 new cases of skin melanoma with 7,990 patients dying of the disease in the US (2). Moreover, it’s thought that there are about 55, 000 deaths globally caused by MM annually (1, 3). While the five-year survival rate for individuals diagnosed with MM ranges from 85% to 93%, the prognosis for older people is much worse (4, 5). Older patients, in particular, require more attention because they have lower immunocompetence and poorer physical conditions, such as concomitant hypertension, diabetes, and other chronic diseases, which prevent them from achieving a favorable prognosis (6, 7). According to the most recent data available from the SEER database, 67.02% of new cases of skin MM in 2019 were in the ≥ 60-year-old age group. In terms of early death, which was defined as an overall survival (OS) time of less than three months after initial diagnosis, there were 6,202 patients recorded in 2019. Among the 6,202 patients, there were 279 patients died within three months from initial diagnosis, and an additional 5,923 patients alive. Of those 279 patients, there were 238 patients ≥ 60 years old, which represents 85.30%, indicating a worse prognosis of skin MM in those older population (8–10).

Facing such a high rate of early death from skin melanoma, the ability to make appropriate risk predictions and take proactive interventions at the time of initial diagnosis has become a major challenge for dermatologists. Currently, the prognosis of early death and the prediction of OS or cancer-special survival (CSS) in patients with skin MM have been described (6–8). The development of an efficient risk prediction model to forecast the risk of early death in the older population is necessary for the intervention and treatment of those older patients at the time of initial diagnosis, but there is currently no study based on big data to investigate which factors contribute to the occurrence of early death in older patients with skin MM. As a simple multivariate oncology visualization tool for forecasting and measuring the survival outcomes of individual patients, the nomogram is already widely acknowledged (11–13). In order to predict the risk of early death in older patients with newly diagnosed skin MM, a diagnostic nomogram was developed and validated in this study using population data from the Surveillance, Epidemiology, and End Results (SEER) database. This research could lead to improved clinical decision-making standards, more judicious use of medical resources, and better patient management for older patients.

## 2 Methods

### 2.1 Patients’ selection

The SEER database, which is based on information from around 28% of the US population, contains clinical pathology and demographic data on cancer incidence and survival rates from numerous medical registries (14). Patients included in this study were those diagnosed as primary skin MM alive or dead within three months from 2000 to 2019 in the SEER database. Through the SEER*Stat program (www.seer.cancer.gov, software version 8.4.3), we were able to access those data [Incidence-SEER Research Plus Date, 17 Registries, Nov 2021 Sub (2000–2019)]. The study is exempt from ethical committee approval and patient informed consent because the data obtained is anonymous and the SEER database is an open-access database.

**Figure 1:**
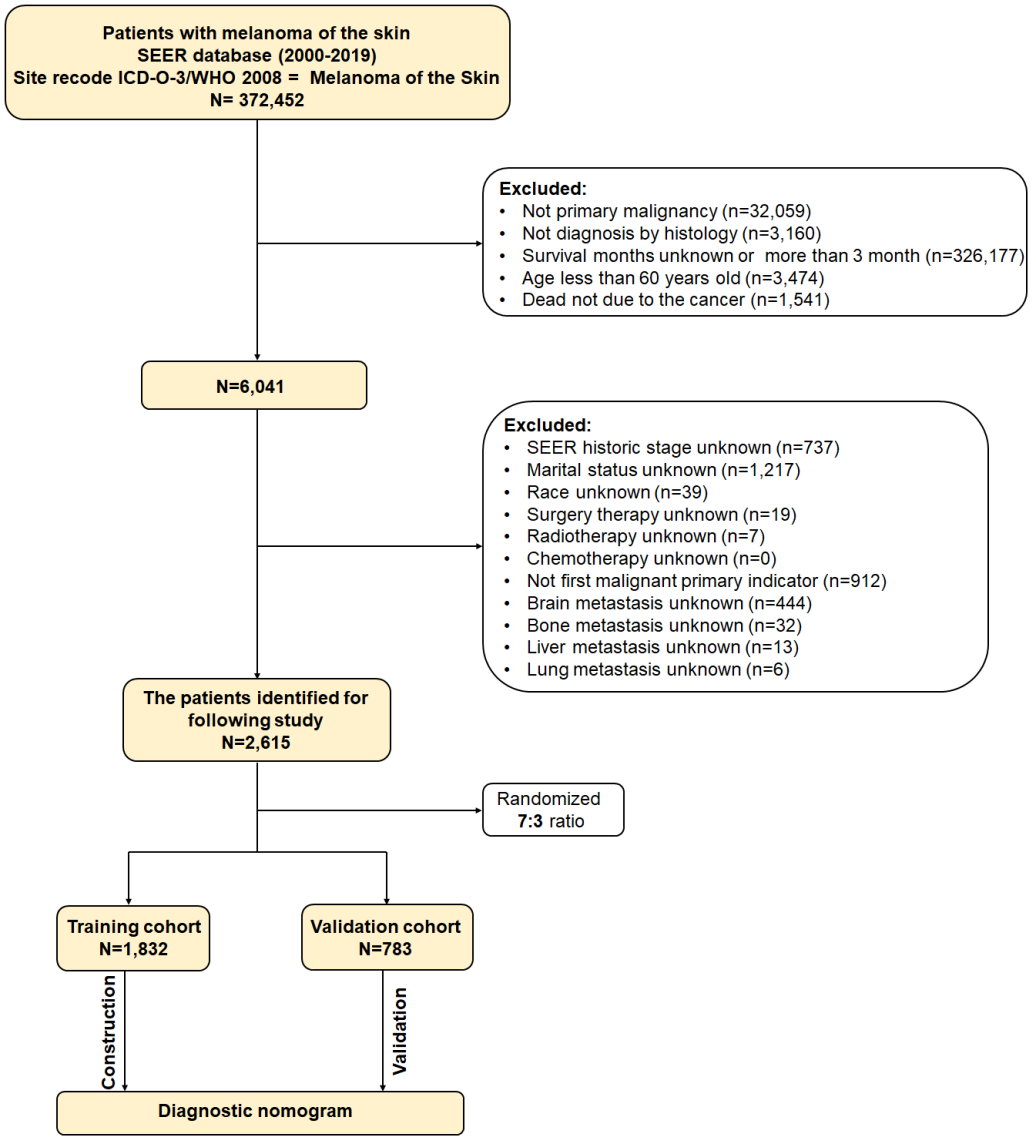
The flow diagram showing how the study’s patients were chosen from the SEER database.

These were the inclusion criteria (1): melanoma of skin as the initial primary tumor (2); age of at least 60 years (15–18) (3); diagnosed by histology (4); the follow-up data is complete. In the meantime, the following were the exclusion criteria (1): the cancer was not the cause of death (2); the survival period was either less than one month or more than three months (3); information about the enrolled variables was unknown. Participants were randomized at random by the RStudio software in a 7:3 ratio between the training and validation cohorts. **Figure 1** displays the flow diagram for this study’s patient selection process.

### 2.2 Variable definitions

Studies vary in their definitions, but generally speaking, early death is characterized as occurring between 30 days and three months following initial diagnosis. Therefore, three months was this study’s definition of early death. This study included 16 variables that might related to early death in older patients with primary skin MM. Age was divided into 60-72, 73-80, and <80 years old based on the X-tile software (19). The definition of other variables was as follows (8): there were divisions for gender (male and female) and race (black, white, and other); married and unmarried (single, unmarried or domestic partner, widowed, separated, and divorced) were the two categories of marital status; median household income was divided into <$50,000, $50,000-74,999, and >$75,000; rural/urban area was divided into non-metropolitan area, <250,000 population, 250,000-1 million population, and >1 million population; tumor stage was divided into localized (tumor confined to the tumor), regional (tumor with direct extension to adjacent organs or structures or spread to regional lymph nodes), and distant (involvement of distant sites or lymph nodes); histological type was divided into MM, nodular melanoma, superficial spreading melanoma, lentigo MM, lentiginous melanoma (Acral/Mucosal), desmoplastic melanoma, and others (rare MM); the primary site was divided into skin (NOS), skin other/unexpected (or unspecified) parts of face, skin of scalp and neck, skin of trunk, skin of upper limb and shoulder, skin of lower limb and hip, and others; distant metastases (bone, brain, liver, and lung) were divided into present and absent; treatment (surgery therapy, radiotherapy, chemotherapy) was divided into yes or no.

### 2.3 Statistical analysis

All statistical analyses of the data in this study were performed using SPSS (version 27.0) and R (version 4.1.0), with a p-value of less than 0.05 being regarded as statistically significant. Using R software, the patients were first randomly randomized at a 7:3 ratio into training and validation groups. In older patients with primary skin MM, the training cohort was utilized to determine independent prognostic risk variables linked to cancer-specific early death and to create a diagnostic nomogram that was later confirmed by the validation cohort. In particular, univariate logistic analysis was performed on the training cohort using SPSS to determine the risk factors associated with cancer-specific early death in those patients. To identify the independent risk factors, variables from the univariate logistic analysis with a p-value <0.05 were then included in the multivariate logistic analysis. Next, utilizing R software, a diagnostic nomogram was created based on those independent risk factors, and in the interim, the factors’ corresponding point assignments were determined (**Supplementary File 1**).

After the nomogram was built, calibration curves were employed to demonstrate the diagnostic nomogram’s agreement with reality. The area under the curve (AUC) by receiver operating characteristic (ROC) curves was obtained to show the diagnostic nomogram’s discrimination ability. In the meantime, each independent risk factors and the diagnostic nomogram’s ROC curve was created to demonstrate its capacity for prediction power using the given AUC value, in which a higher AUC presented a higher prediction power. Ultimately, the clinical usefulness of the nomogram was assessed using a decision curve analysis (DCA).

## 3 Results

### 3.1 Baseline characteristics

2,615 patients who were diagnosed with primary skin MM between 2000 and 2019 were ultimately enrolled in this study based on the inclusion and exclusion criteria. These patients were randomized in a 7:3 ratio into a training group (n = 1,872) and a validation cohort (n = 783). **Table 1** displayed the clinical and pathological characteristics of the individuals that were enrolled.

**Table 1:** The histological and clinical characteristics of primary malignant melanoma of skin in 2,615 older patients.

Variables	Training cohort (1,832)				Validation cohort (783)			
	Alive		Dead		Alive		Dead	
	1318	71.94%	514	28.06%	564	72.03%	219	27.97%
Age (years)								
60-72	764	57.97%	242	47.08%	332	58.87%	111	50.68%
73-80	314	23.82%	126	24.51%	135	23.94%	48	21.92%
>80	240	18.21%	146	28.40%	97	17.20%	60	27.40%
Sex								
Female	503	38.16%	160	31.13%	215	38.12%	71	32.42%
Male	815	61.84%	354	68.87%	349	61.88%	148	67.58%
Race								
Black	4	0.30%	5	0.97%	4	0.71%	4	1.83%
White	1297	98.41%	498	96.89%	553	98.05%	213	97.26%
Other	17	1.29%	11	2.14%	7	1.24%	2	0.91%
Marital status								
Unmarried	408	30.96%	235	45.72%	191	33.87%	105	47.95%
Married	910	69.04%	279	54.28%	373	66.13%	114	52.05%
Median household income								
<$50,000	117	8.88%	66	12.84%	63	11.17%	29	13.24%
$50,000 – $74,999	642	48.71%	279	54.28%	264	46.81%	130	59.36%
>$75,000	559	42.41%	169	32.88%	237	42.02%	60	27.40%
Rural/urban								
Non-metropolitan area	165	12.52%	55	10.70%	66	11.70%	29	13.24%
<250,000 pop	101	7.66%	43	8.37%	54	9.57%	19	8.68%
250,000-1 million pop	276	20.94%	110	21.40%	135	23.94%	58	26.48%
>1 million pop	776	58.88%	306	59.53%	309	54.79%	113	51.60%
Histological types								
Malignant melanoma, NOS	547	41.50%	426	82.88%	233	41.31%	189	86.30%
Nodular melanoma	138	10.47%	38	7.39%	57	10.11%	15	6.85%
Superficial spreading melanoma	456	34.60%	28	5.45%	192	34.04%	7	3.20%
Lentigo malignant melanoma	113	8.57%	2	0.39%	56	9.93%	0	0.00%
Lentiginous melanoma (Acral/Mucosal)	29	2.20%	3	0.58%	11	1.95%	0	0.00%
Desmoplastic melanoma	25	1.90%	4	0.78%	11	1.95%	2	0.91%
Others	10	0.76%	13	2.53%	4	0.71%	6	2.74%
Primary site								
Skin, NOS	49	3.72%	329	64.01%	23	4.08%	160	73.06%
Skin other/unexpected (or unspecified) parts of face	124	9.41%	16	3.11%	64	11.35%	6	2.74%
Skin of scalp and neck	144	10.93%	29	5.64%	66	11.70%	9	4.11%
Skin of trunk	393	29.82%	70	13.62%	169	29.96%	24	10.96%
Skin of upper limb and shoulder	351	26.63%	39	7.59%	133	23.58%	9	4.11%
Skin of lower limb and hip	213	16.16%	25	4.86%	94	16.67%	8	3.65%
Others	44	3.34%	6	1.17%	15	2.66%	3	1.37%
Tumor stage								
Localized	1095	83.08%	32	6.23%	461	81.74%	9	4.11%
Regional	168	12.75%	32	6.23%	72	12.77%	12	5.48%
Distant	55	4.17%	450	87.55%	31	5.50%	199	90.87%
Bone metastasis								
Absent	1303	98.86%	378	73.54%	556	98.58%	160	73.06%
Present	15	1.14%	136	26.46%	8	1.42%	59	26.94%
Brain metastasis								
Absent	1298	98.48%	289	56.23%	557	98.76%	112	51.14%
Present	20	1.52%	225	43.77%	7	1.24%	107	48.86%
Liver metastasis								
Absent	1308	99.24%	348	67.70%	558	98.94%	149	68.04%
Present	10	0.76%	166	32.30%	6	1.06%	70	31.96%
Lung metastasis								
Absent	1286	97.57%	244	47.47%	548	97.16%	98	44.75%
Present	32	2.43%	270	52.53%	16	2.84%	121	55.25%
Surgery therapy								
No	68	5.16%	388	75.49%	35	6.21%	179	81.74%
Yes	1250	94.84%	126	24.51%	529	93.79%	40	18.26%
Radiotherapy								
No	1289	97.80%	315	61.28%	543	96.28%	127	57.99%
Yes	29	2.20%	199	38.72%	21	3.72%	92	42.01%
Chemotherapy								
No	1303	98.86%	452	87.94%	561	99.47%	192	87.67%
Yes	15	1.14%	62	12.06%	3	0.53%	27	12.33%

### 3.2 Identification of independent risk factors

The univariate logistic analysis conducted in the training cohort revealed that the following factors were related to the development of cancer-specific early death in older patients with primary skin MM: age, sex, marital status, median household income, histology, primary site, tumor stage, distant metastases (bone, brain, liver, and lung), and treatment (chemotherapy, radiotherapy, surgery therapy) (p<0.05). Subsequently, the aforementioned variables were subjected to multivariate logistic regression analysis. The findings indicated that the independent risk factors for cancer, particularly for early death in older patients with primary skin MM, were age, histology, liver metastasis, tumor stage, surgery therapy, and radiotherapy (**Table 2**).

**Table 2:** The independent risk factors for early death in older adults with primary malignant melanoma of skin were analyzed using logistic regression.

Variables	Univariate analysis		Multivariate analysis	
	HR (95% CI)	P value	HR (95% CI)	P value
Age (years)				
60-72	Reference		Reference	
73-80	1.27 (0.98-1.63)	0.066	1.13 (0.67-1.9)	0.657
>80	1.92 (1.49-2.47)	<0.001	4.46 (2.65-7.5)	<0.001
Sex				
Female	Reference			
Male	1.37 (1.1-1.7)	0.005		
Race				
Black	Reference			
White	0.31 (0.08-1.15)	0.079		
Other	0.52 (0.11-2.36)	0.395		
Marital status				
Unmarried	Reference			
Married	0.53 (0.43-0.66)	<0.001		
Median household income				
<$50,000	Reference			
$50,000 – $74,999	0.77 (0.55-1.07)	0.125		
>$75,000	0.54 (0.38-0.76)	<0.001		
Rural/urban				
Non-metropolitan area	Reference			
<250,000 pop	1.28 (0.8-2.04)	0.307		
250,000-1 million pop	1.2 (0.82-1.74)	0.353		
>1 million pop	1.18 (0.85-1.65)	0.322		
Histological types				
Malignant melanoma, NOS	Reference		Reference	
Nodular melanoma	0.35 (0.24-0.52)	<0.001	1.2 (0.63-2.29)	0.57
Superficial spreading melanoma	0.08 (0.05-0.12)	<0.001	0.52 (0.27-1)	0.049
Lentigo malignant melanoma	0.02 (0.01-0.09)	<0.001	0.31 (0.07-1.45)	0.137
Lentiginous melanoma (acral/mucosal)	0.13 (0.04-0.44)	0.001	0.48 (0.08-2.9)	0.424
Desmoplastic melanoma	0.21 (0.07-0.59)	0.004	0.48 (0.06-3.58)	0.471
Others	1.67 (0.72-3.84)	0.229	6.31 (1.41-28.15)	0.016
Primary site				
Skin, NOS	Reference			
Skin other/unexpected (or unspecified) parts of face	0.02 (0.01-0.04)	<0.001		
Skin of scalp and neck	0.03 (0.02-0.05)	<0.001		
Skin of trunk	0.03 (0.02-0.04)	<0.001		
Skin of upper limb and shoulder	0.02 (0.01-0.03)	<0.001		
Skin of lower limb and hip	0.02 (0.01-0.03)	<0.001		
Others	0.02 (0.01-0.05)	<0.001		
Tumor stage				
Localized	Reference		Reference	
Regional	6.52 (3.89-10.92)	<0.001	4.56 (2.5-8.3)	<0.001
Distant	279.97(178.63-438.81)	<0.001	76.87 (34.71-170.21)	<0.001
Bone metastasis				
Absent	Reference			
Present	31.25 (18.12-53.92)	<0.001		
Brain metastasis				
Absent	Reference			
Present	50.53 (31.43-81.23)	<0.001		
Liver metastasis				
Absent	Reference		Reference	
Present	62.39 (32.6-119.4)	<0.001	3.24 (1.5-6.99)	0.003
Lung metastasis				
Absent	Reference			
Present	44.47 (30.07-65.76)	<0.001		
Surgery therapy				
No	Reference		Reference	
Yes	0.02 (0.01-0.02)	<0.001	0.23 (0.11-0.47)	<0.001
Radiotherapy				
No	Reference		Reference	
Yes	1.37 (1.1-1.7)	0.005	2.13 (1.14-3.97)	0.017
Chemotherapy				
No	Reference			
Yes	11.92 (6.71-21.15)	<0.001		

### 3.3 Construction and validation of the diagnostic nomogram

**Figure 2:**
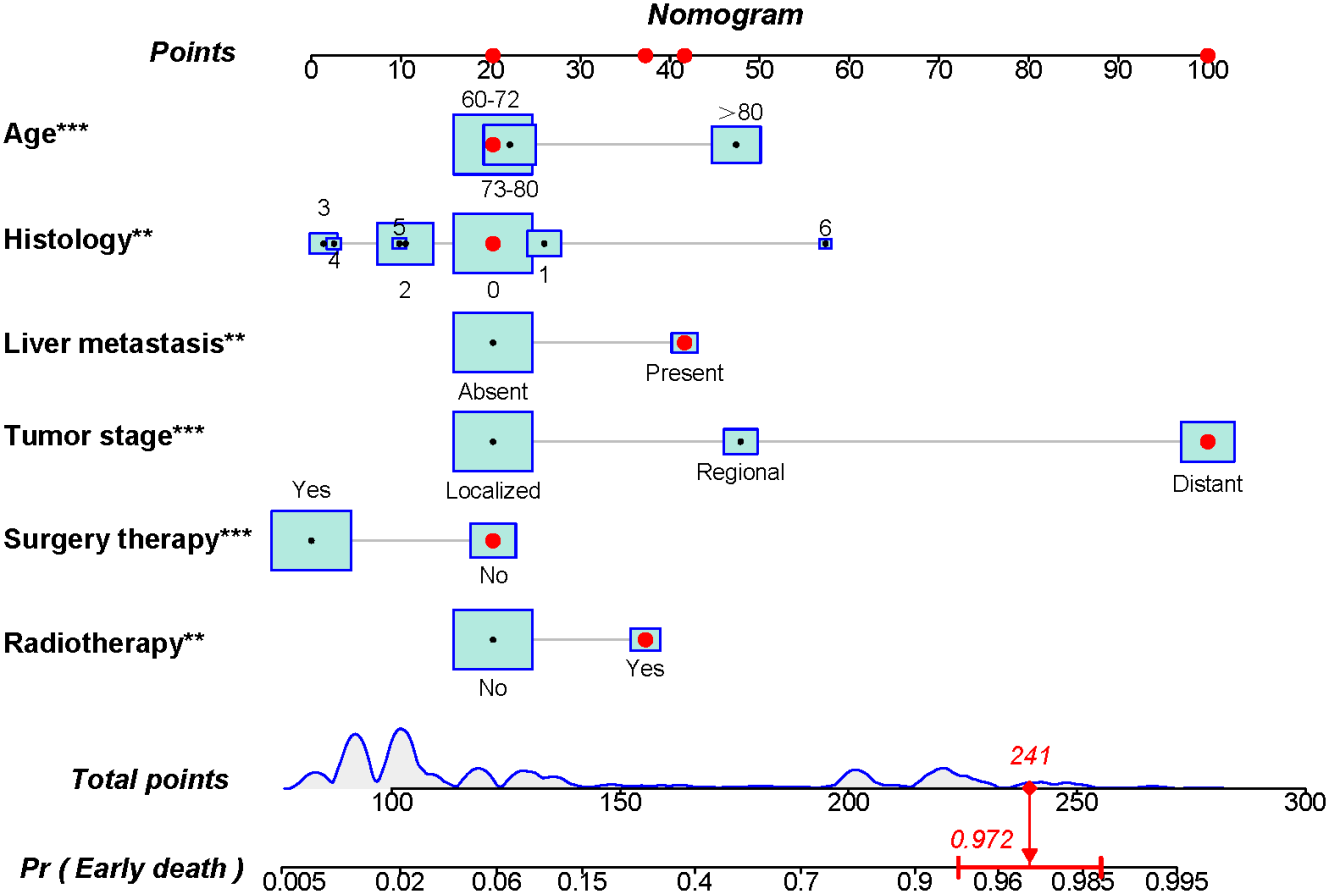
A novel diagnostic nomogram to predict the risk of early death in older patients with primary melanoma of skin. The independent risk factors were age, histology (1: malignant melanoma, NOS; 2: nodular melanoma; 3: superficial spreading melanoma; 4: lentigo malignant melanoma; 5: lentiginous melanoma (acral/mucosal); 6: desmoplastic melanoma; 7: others), liver metastasis, tumor stage, surgery therapy, and radiotherapy). The left variable axis shows the value of each independent risk factor for each patient. A line is then traced upward to the point axis to determine the score given to each independent risk factor, which is then added up to get the total point. In order to calculate the likelihood of early mortality in older primary skin melanoma patients, a vertical line is then drawn from the total score scale to the early death axis. Asterisks indicate p values, **p < 0.05, ***p < 0.001.

**Figure 3:**
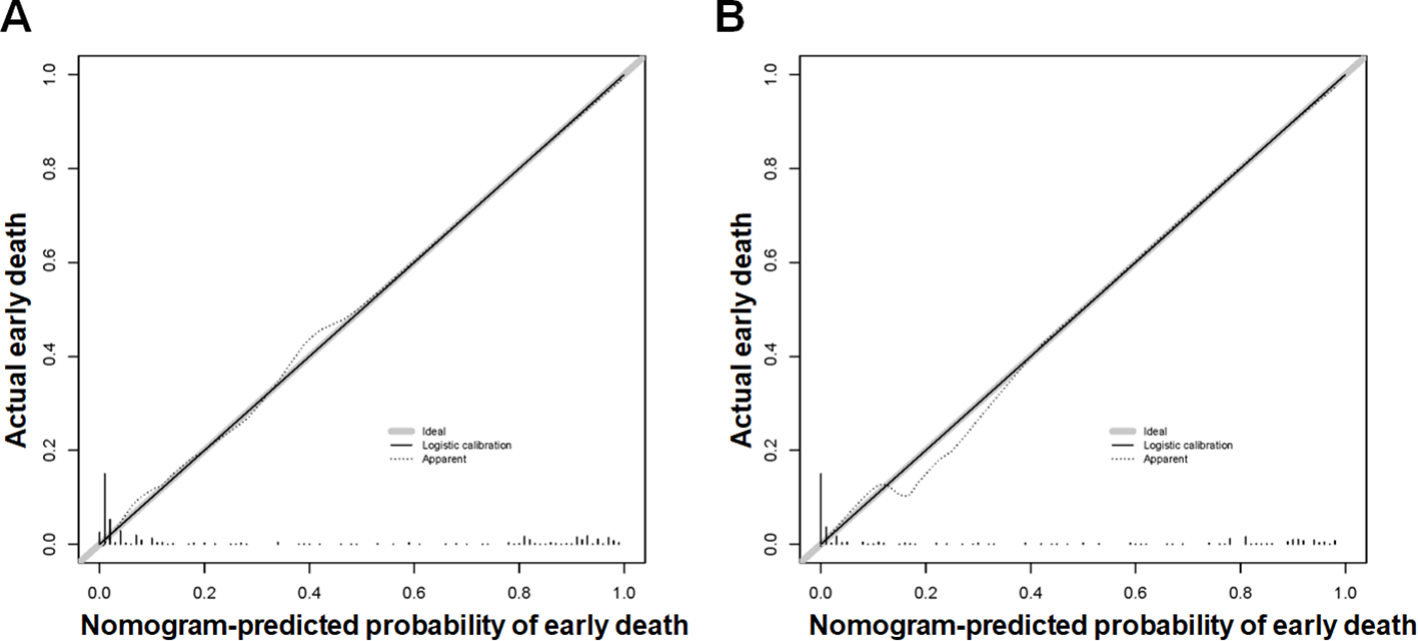
The new diagnostic nomogram’s calibration curves for the training cohort **(A)** and validation cohort **(B)** of older patients with primary melanoma of skin that indicate the likelihood of an early death. The Y-axis shows the actual chance of an early death in older patients with primary melanoma of skin, whereas the X-axis shows the anticipated likelihood of the new diagnostic nomogram of an early death in older patients with primary melanoma of skin. A properly calibrated model, where the expected probability matches the actual values, is represented by the plot along the 45-degree line.

**Figure 4:**
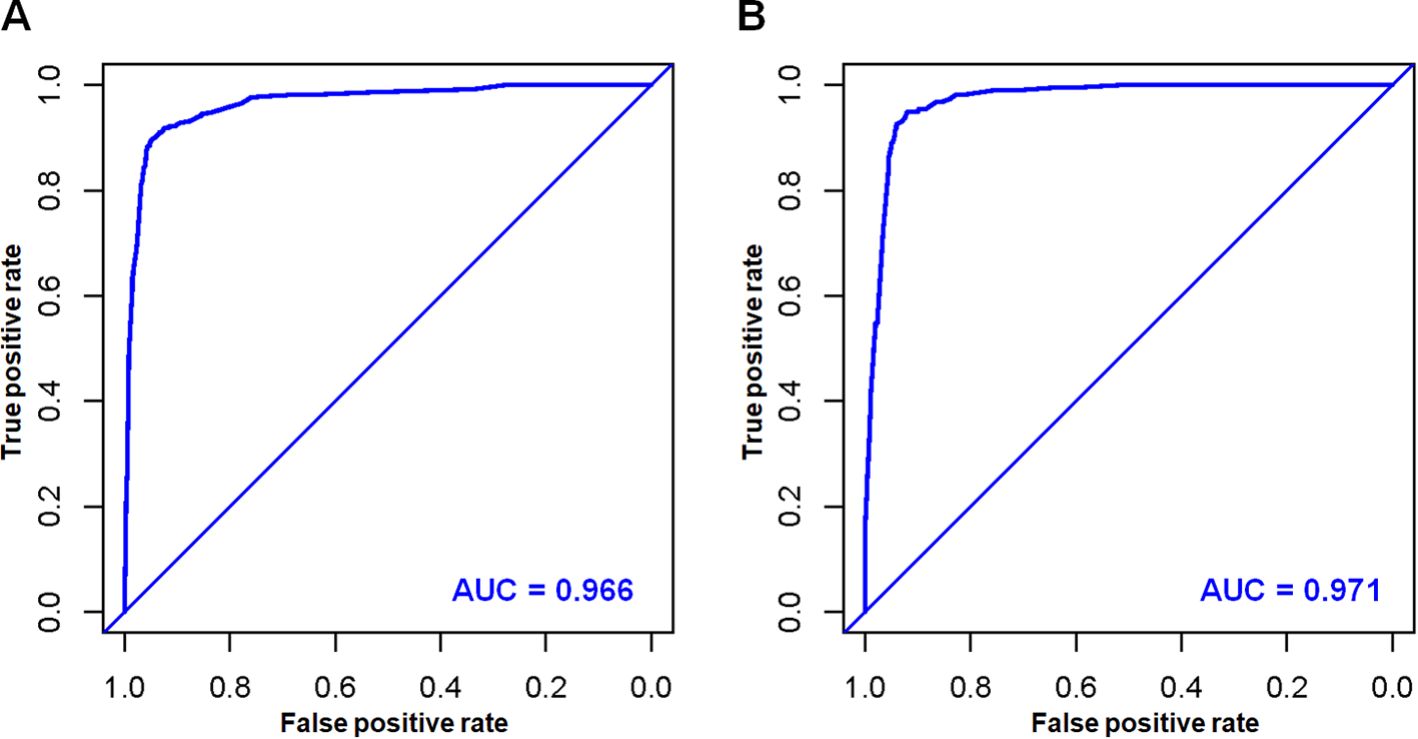
In the training cohort **(A)** and validation cohort **(B)**, the novel diagnostic nomogram’s receiver operating characteristic (ROC) curves and area under the curve (AUC) were used to predict the early death of older patients with primary melanoma of skin.

**Figure 5:**
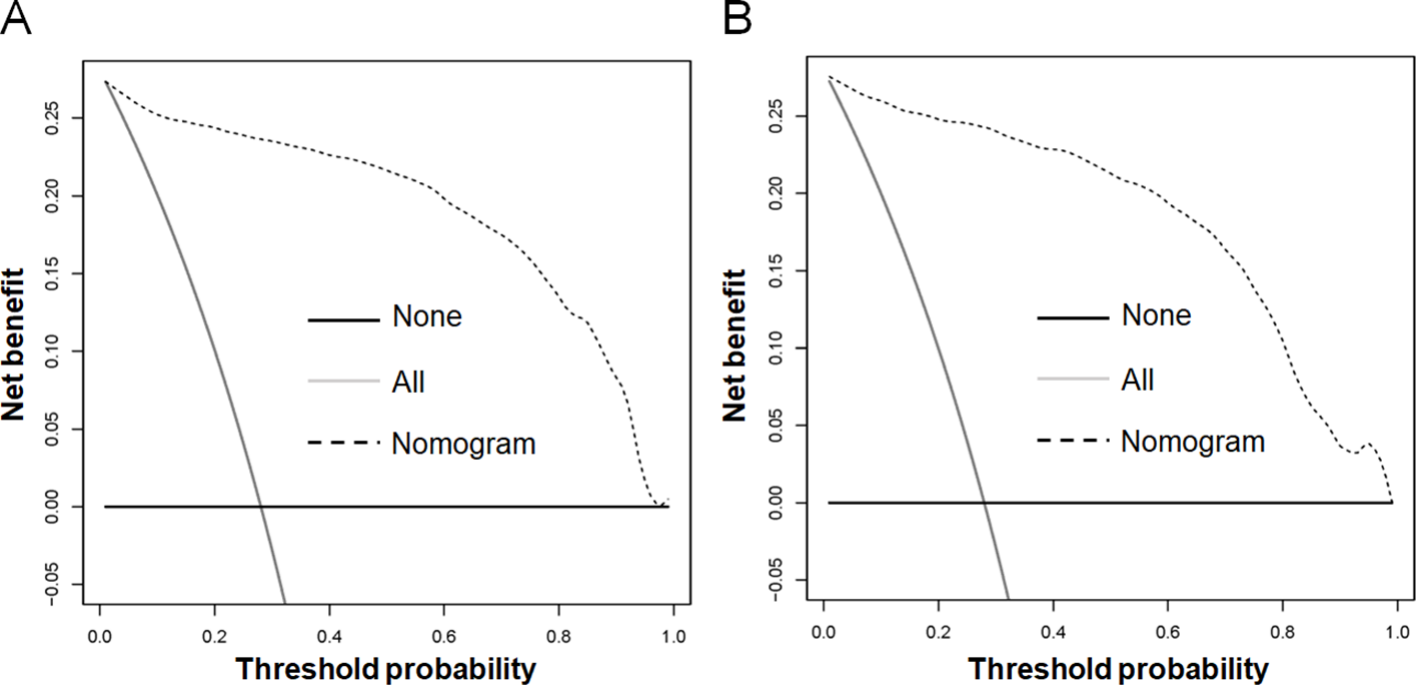
The novel diagnostic nomogram’s decision curve analysis (DCA) was used in the training cohort **(A)** and validation cohort **(B)** to forecast the early death of older patients with primary melanoma of skin.

**Figure 6:**
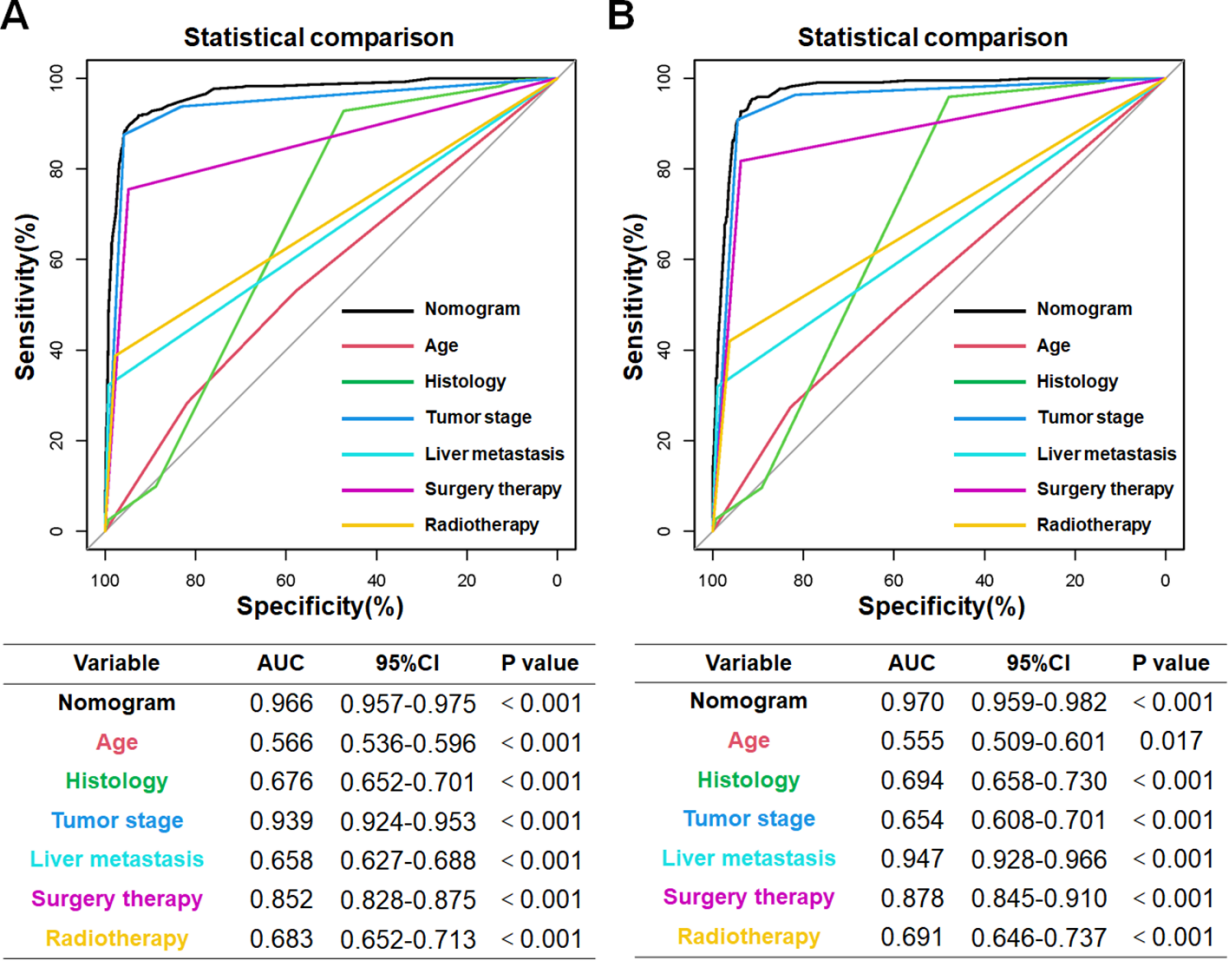
The training cohort **(A)** and validation cohort **(B)** AUC comparison for each independent risk factor pertaining to the innovative diagnostic nomogram. In both the training and validation cohorts, the novel diagnostic nomogram’s AUC was the highest, indicating its exceptional predictive power in forecasting the likelihood of an early death.

Based on the aforementioned six characteristics, a diagnostic nomogram was created to estimate the risk of cancer-specific early death in older patients with primary skin MM (**Figure 2**). The nomogram’s independent risk factors were each given a score based on how much of an impact they had on the result. The values of each independent risk factor for every patient are displayed on the left variable axis. The score assigned to each independent risk factor is then found by drawing a line upward to the point axis, where it is added up to obtain the total point. A vertical line is then drawn from the total point scale to the early death axis to determine the probability of early mortality in older primary skin melanoma patients. There was good agreement between the observed and anticipated probabilities in the calibration curves (**Figure 3**). The training and validation cohorts’ respective AUCs were 0.966 and 0.971 (**Figure 4** **).** In the meantime, the built diagnostic nomogram’s AUC was more than the sum of its individual risk factors (**Figure 5**). The DCA demonstrated the high applicability of the diagnostic nomogram in clinical settings (**Figure 6**).

## 4 Discussion

MM is one of the most aggressive skin tumors, characterized by high occultation, early distant metastasis, and high mortality. Although it accounts for only about 5% of all skin cancers, it causes 75% of skin cancer patients to die (20, 21). MM tends to occur more often in men. According to reports, there are roughly 20 cases of MM for every 100,000 individuals. Among those patients, more than half are in the older population. As the population ages and life expectancy increases, it is anticipated that the prevalence of MM will rise in the older population. According to a report by Seedor and Orloff, the incidence of MM in the older population increased by 1.8% per year between 2009 and 2018, which is more than nine times the incidence in the younger population (22). Moreover, the incidence rate in older men was as high as 4.1% per year (23, 24). Bhatt VR et al. had shown that there are significant differences in the treatment of MM between younger and older populations, younger melanoma patients were more likely to be treated in academic centers, undergo wide resection for stage I–III disease, and receive systemic therapy for stage III–IV disease (25). Therefore, more attention should be devoted to improving survival in the older population.

With the successful development of targeted therapies and immunotherapies for patients with skin MM, older patients’ quality of life and OS have improved significantly (22). However, the mortality rate of MM patients remains high, with approximately 28% of patients in this study dying within three months of diagnosis. To maximize patient survival, more research is required to determine the independent risk factors for early mortality, as there have been few studies evaluating early death in older skin MM patients. Receiving the right treatment and having a better prognosis depend on the early detection of early death at the time of primary skin MM diagnosis. In order to more effectively address this problem, this study used a population-based extensive database to determine the independent risk factors for cancer-specific early death and developed a diagnostic nomogram to assess the likelihood of early death in older primary skin MM patients. To the best of our knowledge, this is the first study to build a novel diagnostic nomogram for those groups and discover independent risk factors.

This study found six independent risk factors, age at diagnosis, tumor stage, histology, liver metastasis, surgery therapy, and radiotherapy, were linked to cancer-specific early death. In addition, a diagnostic nomogram was created and validated in this study in order to forecast cancer-specific early death in older patients with recently diagnosed primary skin MM. There was good agreement between the expected and observed probabilities in the calibration curves. Compared to each independent risk factor, the built diagnostic nomogram’s AUC was greater. Furthermore, the DCA demonstrated the great usefulness of the diagnostic nomogram’s application in clinical settings.

In older patients with primary skin MM, age has been found to be an independent risk factor for cancer-specific early death, and the likelihood of an early death rises with age. On the one hand, cancer treatment in the older population is often complicated by coexisting chronic diseases, cognitive impairment, and social isolation, which may limit access to effective anti-tumor therapy, and complications from postoperative and other therapies can result in patients not receiving effective courses and doses of therapy. On the other hand, an essential factor in the poorer prognosis of the older patients may be a dysregulated anti-tumor immune response. The older the patient, the lower their ability to inhibit tumor growth and delay distant metastasis. Moreover, this study disclosed that among melanoma in the skin, superficial spreading melanoma and lentigo melanoma had better prognoses in terms of histologic subtypes. On the other hand, some rare skin MM, which made up 1.26% of all primary MM in this study, had the worst prognoses, including MM in giant pigmented nevus, epithelioid cell melanoma, melanotic melanoma, and mixed epithelioid and superficial spreading melanoma. Thus, in clinical practice, care should be given to patients who initially come with a rare and unknown skin MM in order to avoid misdiagnosis and treatment delays (26).

Tumor stage and liver metastasis are another two independent risk factors. Patients with distant metastasis had a higher early death possibility than those with local or regional metastasis. With the improvement of diagnostic technology and a series of results achieved by precision medicine in recent years, the survival of skin MM patients has been improved, and some patients without distant metastases may have a five-year survival rate of up to 90% (4, 5). However, the prognosis for patients with advanced skin MM is much worse, with five-year survival rates can be as low as 68% for patients with regional metastatic disease and 11.8% for those with distant metastases, demonstrating the significance of early diagnosis and treatment (4, 27). Moreover, compared to skin MM patients without liver metastases, the survival rate of individuals with liver metastases was much lower. Meanwhile, oncologists might be able to offer more advanced treatment methods, such clinical trials and closer monitoring, to patients who are at a high risk of early death.

Surgery is recommended for the preferred treatment of no metastasis melanoma of skin, allowing for both tumor removal and accurate diagnosis (28, 29). Biopsy surgery could help avoid surgical site errors and clarify the diagnosis and stage of skin melanoma. A wide resection should be the aim of surgical resection in order to minimize the danger of distant metastases or local recurrence, which should be customized to the patient’s needs. In this study, 1,945 (74.38%) patients received surgery. In most circumstances, surgery is still advised for those melanomas with metastases. However, due to the unique biological characteristics of the tumor, patients with certain conditions, such as mucosal melanoma, have a higher chance of experiencing a local recurrence or distant metastasis following surgery than those who do not have metastases at first diagnosis (4, 30).

The benefit of radiotherapy in treating skin MM is not currently well established. MM is considered a radioresistant tumor, but some studies have reported a positive effect of radiotherapy on the survival of specific sites and types of MM. Karasawa et al. treated 23 gynecologic MM patients with inguinal/pelvic lymph node metastases with radiotherapy and achieved a 3-year OS rate of 53% (31). Radiotherapy might be beneficial for patients with positive pathologic margins and positive lymph nodes and be a treatment that can be recommended (32, 33). In contrast, Wu et al.’s study revealed that radiation therapy might not improve a MM patient’s chances of survival, especially those with higher tumor loads and more risk factors. They also warn of the need to be aware of the side effects of radiotherapy (34). According to this study, there was an increased risk of early death in the older population receiving radiation therapy than that in those who did not. We analyzed that, in addition to the naturally occurring resistance to radiotherapy in melanoma of skin, it is also related to the complications brought about by radiotherapy, which to a certain extent impede the possible therapeutic benefits of radiotherapy and make the early mortality rate higher. Therefore, in the face of the special population of older patients, it is necessary to assess the value of the application of radiotherapy and even chemotherapy in all aspects to avoid causing more significant damage to the organism (13).

This study has a number of benefits. First, the study is retrospective study based on a large number of populations. We enrolled 2,615 patients from 372,452 registrants, and after rigorous statistical analysis, the results obtained are of good representative and clinical guidance value. Second, the study’s independent risk factors were readily accessible in standard clinical practice, in contrast to the genetic and molecular level markers linked to primary skin MM. Finally, among older patients with primary skin MM, this is, as far as we know, the first diagnostic nomogram utilized to predict cancer-specific early mortality. Nomogram is a tool that converts complex variable predictions into explicit mathematical assignments, enabling the quantification and calculation of the risk of early mortality.

However, the present study also has several limitations. First, the selection bias was inevitable because it was a retrospective review of the SEER data. Then, many recognized risk factors that may have significantly increased the effectiveness of the current diagnostic nomogram were absent from the SEER database, such as gene mutation data, molecular pathology markers, mitosis, and sentinel lymph node status, which might be relevant to cancer special early death. Finally, the diagnostic nomogram was only validated internally, and more external studies are required to validate the diagnostic nomogram’s usefulness in managing older patients with primary skin melanoma.

## 5 Conclusion

In summary, age, histology, liver metastasis, tumor stage, surgery therapy, and radiation were found to be independent risk factors of cancer, particularly early mortality. These variables were collected from the SEER database along with the clinical and pathological characteristics of the older patient with primary skin MM. A diagnostic nomogram for estimating cancer-specific early death was created and validated using these variables. Given the nomograms’ strong performance, doctors should have no trouble identifying older patients who are most at danger of early death and providing them with specialized care, which will increase their benefit from survival. Undoubtedly, more research is needed to confirm the practical application of this diagnostic nomogram in the management of older patients with primary skin MM.

## Data Availability

The current study’s generated and analyzed dataset from the SEER database can be found at the SEER dataset (https://seer.cancer.gov/). The corresponding author can provide the datasets created and analyzed during the current work upon reasonable request.
