# Supplementary material for: A novel tool for predicting the risk of cancer-specific early death in older patients with primary malignant melanoma of skin: a population-based analysis
Source: Front Oncol. 2024 Sep 6;14:1387014. doi: 10.3389/fonc.2024.1387014 (PMC11412837; doi:10.3389/fonc.2024.1387014)
Supplement: Supplementary file 1 [file Table1.docx]

**Table S1.** The detailed points of independent prognostic risk factors in this nomogram.

| **Variables** | **Corresponding point in the nomogram** |
| --- | --- |
| **Age (years)** | |
| 60-72 | 20 |
| 73-80 | 22 |
| ＞80 | 47 |
| **Tumor stage** | |
| Localized | 20 |
| Regional | 48 |
| Distant | 100 |
| **Histology** | |
| Malignant melanoma, NOS | 20 |
| Nodular melanoma | 26 |
| Superficial spreading melanoma | 11 |
| Lentigo malignant melanoma | 1 |
| Lentiginous melanoma (Acral/Mucosal) | 3 |
| Desmoplastic melanoma | 10 |
| Others | 57 |
| **Liver metastasis** | |
| Absent | 20 |
| Present | 42 |
| **Surgery therapy** | |
| No | 20 |
| Yes | 0 |
| **Radiotherapy** | |
| No | 20 |
| Yes | 37 |
